# Molecular Characterization and Phylogenetic Study of Coxsackievirus A24v Causing Outbreaks of Acute Hemorrhagic Conjunctivitis (AHC) in Brazil

**DOI:** 10.1371/journal.pone.0023206

**Published:** 2011-08-16

**Authors:** Fernando Neto Tavares, Renata de Mendonça Campos, Fernanda Marcicano Burlandy, Rachel Fontella, Maria Mabel Monte de Melo, Eliane Veiga da Costa, Edson Elias da Silva

**Affiliations:** 1 Laboratório de Enterovírus, Instituto Oswaldo Cruz/Fiocruz, Rio de Janeiro, Rio de Janeiro, Brazil; 2 Laboratório de Biologia Evolutiva Teórica e Aplicada, Departamento de Genética, Universidade Federal do Rio de Janeiro, Rio de Janeiro, Rio de Janeiro, Brazil; 3 Laboratório de Virologia - Laboratório Central de Saúde Pública (LACEN), Recife, Pernambuco, Brazil; The University of Hong Kong, Hong Kong

## Abstract

**Background:**

Coxsackievirus A24 variant (CA24v) is the most prevalent viral pathogen associated with acute hemorrhagic conjunctivitis (AHC) outbreaks. Sixteen years after its first outbreak in Brazil, this agent reemerged in 2003 in Brazil, spread to nearly all states and caused outbreaks until 2005. In 2009, a new outbreak occurred in the northeast region of the country. In this study, we performed a viral isolation in cell culture and characterized clinical samples collected from patients presenting symptoms during the outbreak of 2005 in Vitória, Espírito Santo State (ES) and the outbreak of 2009 in Recife, Pernambuco State (PE). We also performed a phylogenetic analysis of worldwide strains and all meaningful Brazilian isolates since 2003.

**Methods and Findings:**

Sterile cotton swabs were used to collect eye discharges, and all 210 clinical samples were used to inoculate cell cultures. Cytopathic effects in HEp-2 cells were seen in 58 of 180 (32%) samples from Vitória and 3 of 30 (10%) samples from Recife. Phylogenetic analysis based on a fragment of the VP1 and 3C gene revealed that the CA24v causing outbreaks in Brazil during the years 2003, 2004 and 2005 evolved from Asian isolates that had caused the South Korean outbreak of AHC during the summer of 2002. However, the 2009 outbreak of AHC in Pernambuco was originated from the reintroduction of a new CA24v strain that was circulating during 2007 in Asia, where CA24v outbreaks has been continuously reported since 1970.

**Conclusions:**

This study is the first phylogenetic analysis of AHC outbreaks caused by CA24v in Brazil. The results showed that Asian strains of CA24v were responsible for the outbreaks since 1987 and were independently introduced to Brazil in 2003 and 2009. Phylogenetic analysis of complete VP1 gene is a useful tool for studying the epidemiology of enteroviruses associated with outbreaks.

## Introduction

Acute hemorrhagic conjunctivitis (AHC) is a highly contagious viral syndrome that frequently causes outbreaks. Transmission occurs primarily via person-to-person contact or contact with contaminated objects (e.g. towels). The disease is self-limiting and is characterized by the sudden onset of ocular pain, eyelid swelling, a foreign body sensation or irritation, epiphora (excessive tearing), eye discharge, and photophobia. In addition, palpebral conjunctival follicular reaction, subconjunctival hemorrhage, and congestion are common. The symptoms appear after a short incubation period of 12 to 48 hours, and the clinical signs typically disappear within 1 to 2 weeks [1]–[3].

Although initially recognized in 1969 to be caused by enterovirus 70 (EV70), AHC is most frequently caused by a variant of coxsackievirus A24 (CA24v) and less frequently by adenoviruses. CA24v is an antigenic variant of the CVA24 strain. EV70 and CA24v are classified as members of the Human Enterovirus C species (HEV-C) and were first isolated from an AHC outbreak that occurred in 1969 and 1970 respectively in Asia [2].

In recent years, CA24v has been implicated as the major causative agent of AHC outbreaks in many countries [1], [3]–[15], including Brazil where this agent was first detected in 1987 [16], [17].

**Figure 1:**
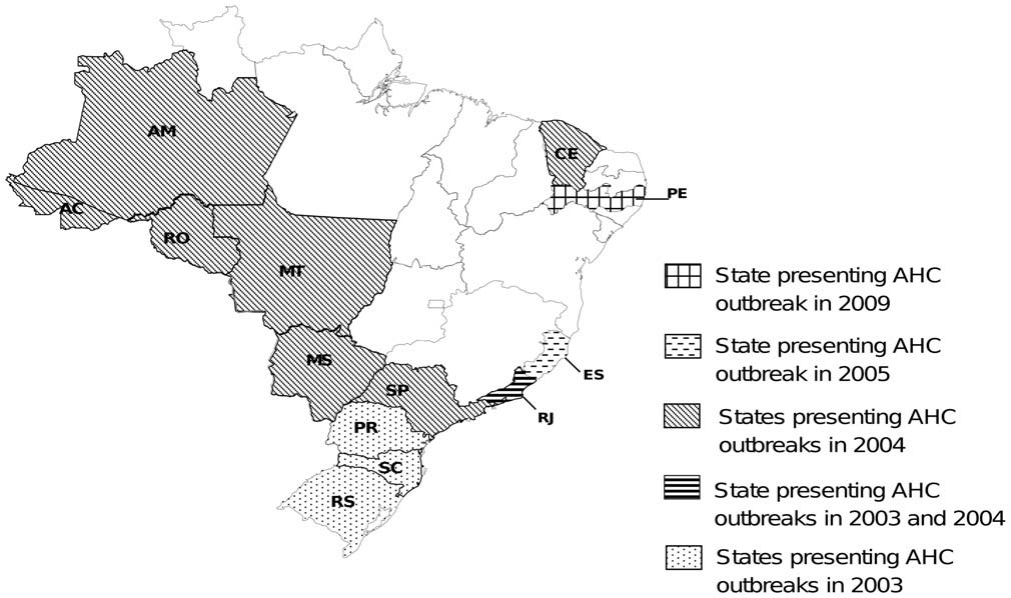
Geographic distribution of CA24v outbreaks in Brazil, 2003–2009.

AHC caused by CA24v reemerged in Brazil in February 2003, sixteen years after the first outbreak. The first 2003 cases occurred in Southern Brazil (Rio Grande do Sul and Santa Catarina States), and subsequent cases were reported in Paraná, São Paulo, Mato Grosso, Mato Grosso do Sul, Rondônia, Acre, Amazonas, Ceará and Rio de Janeiro (Fig. 1). By April of 2003, more than 200,000 cases had been officially reported [9], [18]. Other AHC outbreaks were reported in the country as follows: in 2004, Rio de Janeiro reported more than 60,000 cases in just over 2 months at the beginning of the epidemic [12]; in 2005, there was an outbreak in Vitória, Espírito Santo State (unpublished data); and in 2009, the city of Recife, Pernambuco State (northeast Brazil) reported more than 11,000 cases in just one of the state public health hospitals in May. No AHC cases temporally associated with these outbreaks were so far reported in other countries of South America.

We report the first phylogenetic study of AHC caused by CA24v. We analyzed the VP1 and 3C regions of strains circulating in Brazil between 2003 and 2009. We also determined and analyzed the sequence of the virus responsible for the first outbreak in Brazil in 1987. Data obtained were also compared with known VP1 and 3C sequences from worldwide outbreaks to establish the genetic relationships among CA24v worldwide. Additionally, our data facilitates the production of a data bank for future analyses.

## Results

### Viral Isolation and Molecular Typing of the Isolates

From the 180 conjunctival swabs received in 2005 during the AHC outbreak in Vitória, ES and 30 samples received from the 2009 outbreak in Recife, 58 samples (32%) and 3 samples (10%), showed CPE in HEp-2 cells, respectively. The RD cell line did not support the replication of the virus. Sequence analysis of the VP1∼350 bp fragment identified CA24v as the causative agent of the AHC outbreaks in Vitória/ES and Recife/PE that occurred during the autumns of 2005 and 2009, respectively.

### Direct RNA Detection in Clinical Specimens

Due to the low viral isolation rate obtained from the cell cultures (3 positives out of 30 tested), the 27 clinical specimens from the 2009 AHC outbreak that remained negative for isolation after three HEp-2 passages were used for RNA extraction and RT-PCR for human enterovirus [19]. Nine (33%) of them showed PCR products of approximately 350 bp. Nucleotide sequences of the PCR products were compared with those available at GenBank by using the BLAST Software [20] and showed the presence of CA24v in the samples.

### Phylogenetic Analysis of the VP1 and 3C Genes

Sequences from the CA24v Brazilian isolates were compared to those available in GenBank. In their 3C gene sequences, viral isolates from the outbreaks showed >98% identity (2003 and 2004) and >97% identity (2005) with samples from the 2002 outbreak in Korea. Isolates from the 2009 outbreak in Recife in northeast Brazil showed >98% identity with CA24v viruses isolated in Taiwan in 2006/2007.

**Figure 2:**
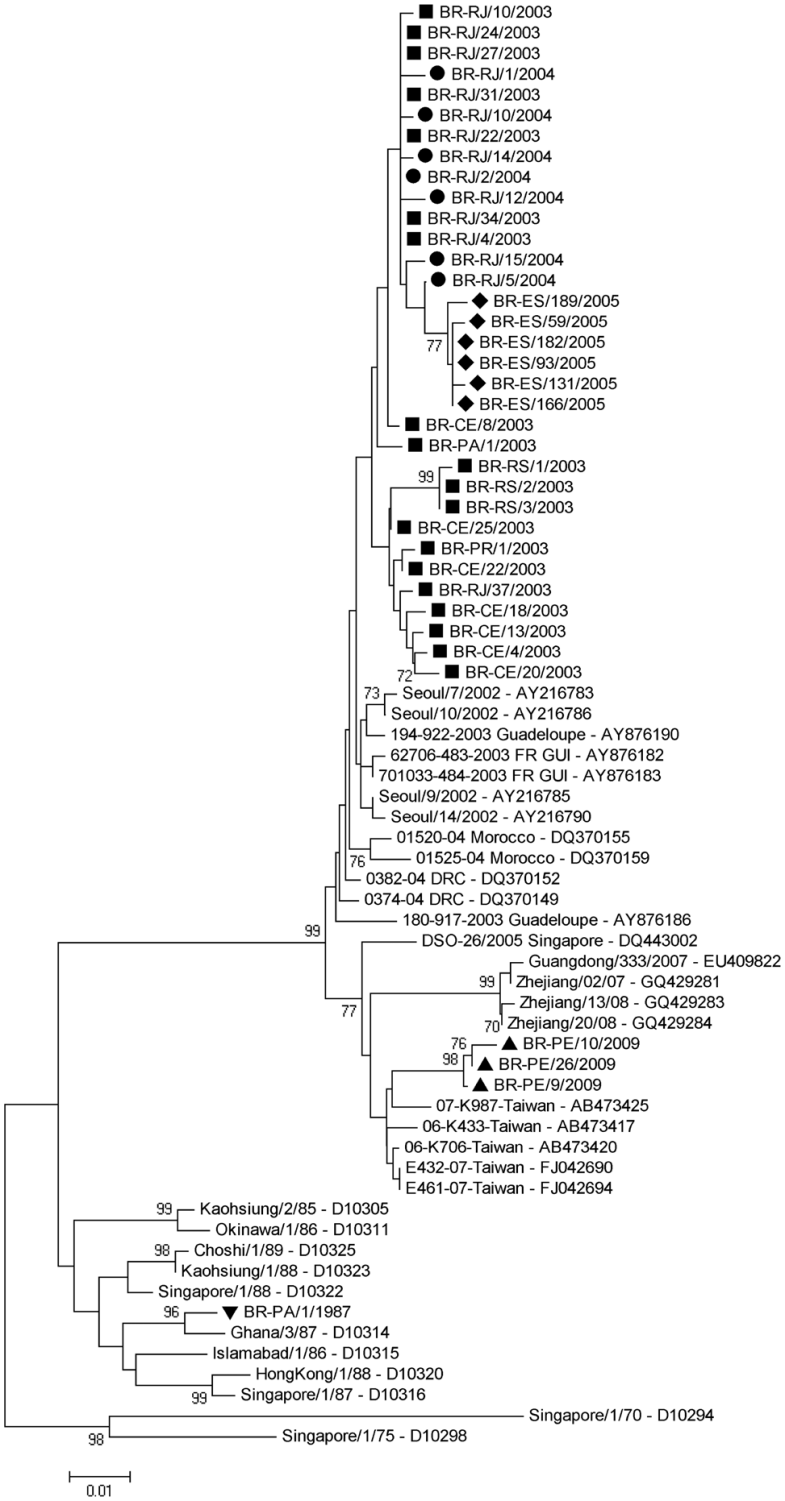
Phylogenetic analysis based on the 510-nucleotide 3C gene. This phylogenetic tree was constructed by the neighbor joining method using the Kimura-two parameter model from MEGA4 software, with 1000 replicates. Thirty-seven Brazilian isolates of CA24v were compared to strains reported from other countries in previous years available in GenBank. Only bootstrap values >70% are shown at the node. Geometric shapes indicate the years of the Brazilian isolates.

Phylogenetic analysis of the 510 nucleotides of the 3C gene was performed with the 37 isolates from Brazil and 33 isolates from different parts of the world that were available in GenBank (Fig. 2). Isolates from the outbreaks of 2003, 2004 and 2005 were closely related to the strains isolated in Korea in 2002. Isolates from 2009 were clustered and closely related to isolates from the 2006/2007 Taiwanese outbreak. The isolate from the first outbreak of CA24v reported in Brazil in 1987 was closely related to the CA24v that caused an outbreak in Ghana during the same year. The two strains displayed >98% identity.

**Figure 3:**
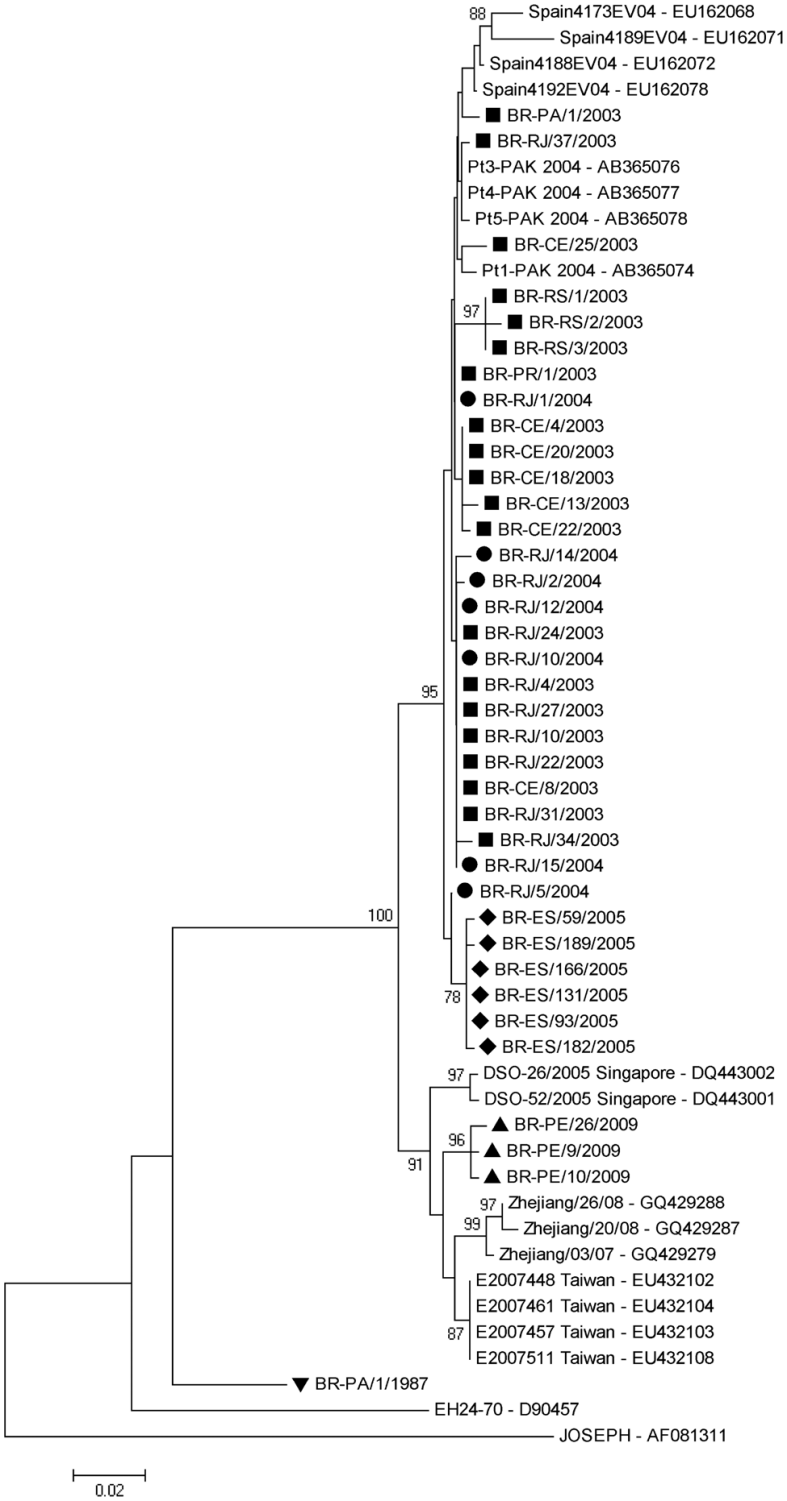
Phylogenetic analysis based on a 473-nucleotide region of the VP1 gene. This phylogenetic tree was constructed by the neighbor joining method using Kimura-two parameter model from MEGA4 software, with 1000 replicates. Thirty-seven sequences were compared to those strains reported from others countries in previous years available in GenBank. Only bootstrap values >70% are shown at the node. Geometric shapes indicate the years of the Brazilian isolates.

Due to the unavailability of complete VP1 sequences in GenBank, phylogenetic analysis was performed using a 473-nucleotide region of this gene for all 37 Brazilian isolates. Nineteen worldwide isolates available at GenBank were also included (Fig. 3). Phylogenetic analysis using the complete 915-nucleotide VP1 gene for Brazilian isolates showed a better temporal clustering than the partial VP1 sequence of 473 nucleotides (data not shown).

All VP1 sequences from the 2003, 2004 and 2005 Brazilian outbreaks were clustered together with the CA24v isolated in Spain and Pakistan during 2004. Isolates from Pernambuco in 2009 showed a close relationship with CA24v from Taiwan 2007 (Fig. 3). The 2009 Pernambuco isolates shared 97.6% to 98.6% identity in 3C and 98.3% to 98.5% identity in the partial VP1 sequence with the 2007 Taiwanese strain.

Isolates from the 2005 outbreak in Espírito Santo in were clustered in both VP1 and 3C in a separate cluster within the group of isolates from the 2003 and 2004 outbreaks in Brazil.

## Discussion

The first outbreak of AHC caused by a variant of CA24v was described in Singapore in 1970 [21]. From 1970 to 1984, the disease appeared to be restricted to southwest Asia and India. However, since 1985, several AHC outbreaks caused by CA24v have been reported around the world, and the virus has been detected in Japan, Taiwan, Oceania, Central America, South America and Africa [1]–[3], [8], [10]–[14], [22].

CA24v was first identified in Brazil in 1987 as the causative agent of an outbreak of AHC in Belém, Pará State. Although temporally related to (>98% identity) and sharing a common ancestor with the CA24v isolated in Ghana [23] in the same year, it is not possible to infer the direct origin of this first recognized Brazilian strain of CA24v.

In February 2003, CA24v reemerged in Brazil to cause the largest known AHC epidemic, with more than 200,000 cases officially reported over most regions of the Brazilian territory [9], [18]. Because AHC reporting is not mandatory, it is likely that the actual number of cases was much higher; most cases were reported to the Health State Departments only at the beginning of the epidemic. This outbreak was subsequently reported in French Guiana, Puerto Rico, and the Caribbean Islands [9], [12], [18]. Based on the phylogenetic analysis of nucleotide sequences (Fig. 2, 3), the CA24v strains in circulation in Brazil from 2003 appear to share the same ancestor with strains causing subsequent outbreaks reported in the Republic Democratic of Congo, Morocco, Spain and Pakistan [10], [13], [14].

The present study also demonstrated that CA24v was the causative agent of the AHC outbreak in Recife, Pernambuco State in the autumn of 2009. Sequence analysis performed on 3 isolates showed >99% identity in their VP1 and 3C genes, which would be consistent with a common source of this outbreak. Phylogenetic analysis also revealed that the isolates from the 2009 outbreak were closely related to isolates from the 2006/2007 AHC outbreak in Taiwan.

Sequence information obtained from the analysis of the VP1 gene has been largely utilized for molecular epidemiologic studies of enteroviruses. This gene contains serotype-specific information and stable and variable domains that can be used for virus identification and evolutionary studies [24], [25].

Due to the limited number of complete VP1 and 3C sequences of CA24v available in GenBank, we used a 473-nucleotide region of VP1 and 510-nucleotide of 3C, which correspond to the vast majority of the available CA24v sequences. In this manner, it was possible to perform the phylogenetic analysis of a reasonable number of sequences representative of several countries. This was particularly useful for the analysis of CA24v isolates from previous outbreaks in Asian countries for which complete VP1 sequences were not available.

Results based on protease 3C demonstrated that all Brazilian isolates, except the Pernambuco ones from 2009, were closely related and formed a major cluster with isolates from South Korea, French Guiana, Guadeloupe, Democratic Republic of Congo, and Morocco. Our phylogenetic analysis suggests a common source for these isolates, most likely to be the recent Asian lineage of a CA24v that appeared in South Korea in 2002 before spreading to other countries.

Our data suggest that the CA24v that circulated within Brazil during 2003, 2004 and 2005 evolved from the previous Asian isolates that caused the South Korean outbreak of AHC during the summer of 2002. However, the 2009 outbreak of AHC in Pernambuco was originated from the reintroduction of a new CA24v strain that was circulating during 2007 in Asia, where CA24v outbreaks has been continuously reported since 1970.

Although other studies of AHC outbreaks have routinely used the 3C gene for molecular analysis, data from analysis of this region are consistent with those using the main protein of the capsid encoded by the VP1 gene. Several studies have used the VP1 gene to determine serotype and shown that this region is more informative for molecular epidemiological studies than the 3C gene. This is because of high recombination rates among enteroviruses, which could distort the analysis, and serological pressure on the capsid region, which could therefore evolve faster than the region encoding non-structural proteins. As we expected, the phylogenetic analysis based on the complete VP1 gene from Brazilian isolates showed a better temporal distribution (clearly differentiated between 2003 and 2004) than analysis based on the partial VP1 sequence. Accordingly, complete sequencing of this region would be of great value for such studies.

## Materials and Methods

### Ethics Statement

The Enterovirus laboratory at FIOCRUZ is an official Brazilian Ministry of Health Reference Laboratory. Coxsackieviruses A24 isolates utilized in the present study are part of the virus collection of the laboratory. During the outbreaks patients voluntarily attended to the local public health system ambulatories where the clinical samples (tears) were collected. This activity was considered as a public health response to the acute hemorrhagic conjunctivitis outbreaks and thus did not require review by the review board. Besides all data was analyzed anonymously.

### Clinical Specimens and Virus Isolation

Sterile cotton swabs were used to collect eye discharges from AHC patients in the cities of Vitória/ES in 2005 and of Recife/PE in 2009. Each swab was immersed in 1 mL viral transport medium (VTM; 1× Hank's balanced salt solution, pH 7,4 containing 5% fetal bovine serum, 100 U/mL penicillin, and 100 µg/mL streptomycin) in 5 mL cryovials. All clinical samples were kept on dry ice or at −70°C and transported to the Enterovirus Laboratory at Fiocruz, Rio de Janeiro, Brazil.

Two hundred microliters of each clinical sample were used to inoculate HEp-2 and RD cell lines. Cells were incubated at 37°C and examined daily for cytopathic effect (CPE). Culture supernatants showing CPE were collected and frozen at −20°C. Two blind passages, seven days apart, were performed when no CPE was observed during the first passage.

### Molecular Typing of the Isolates

Viral RNA was extracted by using Trizol LS (Invitrogen), according to the manufacturer's instructions, from the culture supernatants that showed CPE and used as a template for cDNA construction with random primers (Promega, Madison, USA) and SuperScript III Reverse Transcriptase (Invitrogen, Carlsbad, CA, USA) following the manufacturer's instructions. PCR was performed using a primer pair (222 and 292) that amplifies a fragment of approximately 350 bp within the VP1 gene [19]. After 35 cycles (94°C for 30 s, 42°C for 30 s, and 60°C and 30 s) in a model 9700 thermocycler (Applied Biosystems, Foster City, CA, USA), PCR products were analyzed by electrophoresis in 1% agarose gels containing 0.5 µg/mL ethidium bromide and compared to a 50 bp DNA Ladder (Invitrogen) by visualization with an UV transilluminator. Specific products were gel purified with the QIAquick Gel Extraction Kit (QIAGEN, Hilden, Germany) and quantified by comparison with the Low DNA Mass Ladder (Invitrogen) in a 1% agarose gel. Sequencing reactions were performed by using the ABI PRISM BigDye Terminator v3.1 Cycle Sequencing Ready Reaction Kit (PE Applied Biosystems, Foster City, CA, USA) in a GeneAmp PCR System 9700 thermocycler (Applied Biosystems, Foster City, CA, USA) with 25 cycles of 96°C for 15 s, 42°C for 30 s, and 60°C for 3 min. Products were purified by isopropyl alcohol precipitation, and samples were analyzed using the ABI PRISM 310 Genetic Analyzer (Applied Biosystems). Partial VP1 sequences from our isolates were compared using BLAST software to those available in GenBank to determine viral serotypes [20].

### Direct Detection of Enterovirus Genome by RT-PCR

Viral RNA was extracted from clinical samples obtained during the 2009 outbreak by using Trizol LS according to the manufacturer's instructions. RT-PCR and cycle sequencing reactions were performed as described above.

### Complete Nucleotide Sequencing of the VP1 and 3C Genes

Thirty-seven samples were sequenced and used for phylogenetic analysis (Table 1). Complete nucleotide sequences of the VP1 and 3C genes were obtained by using specific primers complementary to these regions (Table 2). Products of 1096 bp and 673 bp were obtained from PCR reactions using primers pairs that flank the VP1 and 3C genes, respectively. PCR reaction mixtures of 50 µL contained: 3 µL cDNA, 5 µL 10× PCR buffer, 1.5 mM MgCl_2_, 0.6 µmol/L of each primer (VP1R/VP1F and 3CR/3CF), 2.5 units of Platinum Taq DNA Polymerase (Invitrogen), and 0.5 mM each dNTP (dATP, dCTP, dGTP, dTTP (Invitrogen). Reactions were incubated at 95°C for 2 min, followed by 35 cycles of 95°C for 30 s, 50°C for 30 s and 70°C for 2 min. After the last cycle, polymerization was continued at 72°C for 5 min. Products were analyzed by electrophoresis in a 1% agarose gel containing ethidium bromide (0.5 µg/mL) using a 100 bp DNA Ladder (Invitrogen) and visualized in a UV transilluminator. Specific products were gel purified using the QIAquick Gel Extraction Kit (QIAGEN) and then quantified by comparison with the Low DNA Mass Ladder (Invitrogen) in a 1% agarose gel. Sequencing reactions (25 cycles at 96°C for 30 s, 50°C for 30 s and 70°C for 2 min) were performed with the same sets of primers in both strands. Products were purified and analyzed using the ABI PRISM 310 Genetic Analyzer as described above.

**Table 1:** Origin and number of CA24v isolates from Brazilian AHC outbreaks that were used for phylogenetic study.

State	Year	Sample received	Sample isolated in cell culture	Number of samples sequenced
Pará (PA)	1987	1	*	1
Ceará (CE)	2003	29	11	7
Pará (PA)	2003	1	*	1
Paraná (PR)	2003	3	1	1
Rio de Janeiro (RJ)	2003	37	13	8
Rio Grande do Sul (RS)	2003	3	*	3
Rio de Janeiro (RJ)	2004	15	8	7
Espírito Santo (ES)	2005	180	58	6
Pernambuco (PE)	2009	30	3	3

*Samples received previously isolated in cell culture.

**Table 2:** Oligonucleotide primer sequences for VP1 and 3C amplification of CA24v.

Primer	Nucleotide sequence (5′ – 3′)	Nucleotide position*
**VP1R**	CGG CAT TGC TGT GGT CTT CA	3480–3499
**VP1F**	CTT TGT GAG TGC TTG CCC GG	2403–2422
**3CR**	ACT TCT TTT GAT GGT CTC AT	6025–6044
**3CF**	TCA AAA CTG TTT GCT GGG CA	5371–5390

*Nucleotide position according CA24v reference strain EH24/70.

### Sequence Edition and Phylogenetic Analysis

The nucleotide sequences of VP1 and 3C were aligned using the CLUSTAL W program included in the BioEdit version 7.0.9.0 Software [26] and hand edited where necessary. The alignment was used to identify respective divergence and to infer the phylogenetic relationships among the Brazilian isolates and sequences available in GenBank from outbreaks that had occurred in other parts of the world.

Sequence analyses were performed using MEGA software version 4.0 [27], and the phylogenetic trees were constructed by the neighbor-joining method. The evolutionary distances were computed using the Kimura-two parameter model of nucleotide substitution. The robustness of each node was assessed by a bootstrap test with 1000 replicates.

### Nucleotide Sequence Accession Numbers

The complete sequences of VP1 (915 bp) and 3C (549 bp) from this study are available in GenBank with the respective accession numbers of GU983170 to GU983206 and GU983207 to GU983243.
